# *Smad4 *haploinsufficiency: a matter of dosage

**DOI:** 10.1186/1755-8417-1-2

**Published:** 2008-11-03

**Authors:** Paola Alberici, Claudia Gaspar, Patrick Franken, Marcin M Gorski, Ingrid de Vries, Rodney J Scott, Ari Ristimäki, Lauri A Aaltonen, Riccardo Fodde

**Affiliations:** 1 Department of Pathology, Josephine Nefkens Institute, Erasmus MC, Rotterdam, The Netherlands; 2 Department of Biochemistry, Erasmus MC, Rotterdam, The Netherlands; 3 Newcastle Bowel Cancer Research Collaborative, Hunter Medical Research Institute, John Hunter Hospital and The University of Newcastle, Newcastle, Australia; 4 Division of Pathology HUSLAB and Haartman Institute, Helsinki University Central Hospital, Helsinki, Finland; 5 Department of Medical Genetics, HUSLAB and Haartman Institute, Helsinki University Central Hospital, Finland; 6 Genome Scale Biology Program, Biomedicum Helsinki, University of Helsinki, Helsinki, Finland; 7 Current address: IFOM -The FIRC Institute of Molecular Oncology, IFOM-IEO Campus, Milano, Italy; 8 Current address: Department of Experimental Oncology, European Institute of Oncology (IEO), IFOM-IEO Campus, Milano, Italy

## Abstract

**Background:**

The inactivation of tumor suppressor genes follows Alfred Knudson's 'two-hit' model: both alleles need to be inactivated by independent mutation events to trigger tumor formation. However, in a minority of tumor suppressor genes a single hit is sufficient to initiate tumorigenesis notwithstanding the presence of the wild-type allele, a condition known as haploinsufficiency. The *SMAD4 *gene is an intracellular mediator of the TGF-β and BMP signal transduction pathways and a tumor suppressor involved in pancreatic and colorectal tumorigenesis. In *Smad4*-mutant mouse models, haploinsufficiency characterizes the development of gastrointestinal polyps with initial retention of the wild-type allele and protein expression within the nascent tumors and in their direct microenvironment. Similarly, germline *SMAD4 *mutations are responsible for a subset of patients affected by juvenile polyposis syndrome, an autosomal dominant intestinal cancer syndrome. To date, the molecular and cellular consequences of *SMAD4 *haploinsufficiency on TGF-β and BMP signaling and on genome-wide gene expression have not been investigated.

**Results:**

Here we show that, similar to previous observations in *Smad4*-mutant mouse models, haploinsufficiency characterizes a substantial fraction of the juvenile polyps arising in patients with germline *SMAD4 *mutations. Also, mouse embryonic and intestinal cells heterozygous for a targeted *Smad4 *null mutation are characterized by a corresponding 50% reduction of the Smad4 protein levels. Reporter assays revealed that mouse *Smad4*^+/- ^cells exert intermediate inhibitory effects on both TGF-β and BMP signaling. Genome-wide expression profiling analysis of *Smad4*^+/- ^and *Smad4*^-/- ^cells pinpointed a subset of dosage-dependent transcriptional target genes encompassing, among others, members of the TGF-β and Wnt signaling pathways. These SMAD4 dosage-dependent transcriptional changes were confirmed and validated in a subset of target genes in intestinal tissues from juvenile polyposis syndrome patients.

**Conclusion:**

*Smad4 *haploinsufficiency is sufficient to significantly inhibit both TGF-β and BMP signal transduction and results in the differential expression of a broad subset of target genes likely to underlie tumor formation both from the mesenchymal and epithelial compartments. The results of our study, performed in normal rather than tumor cells where additional (epi-) genetic alterations may confound the analysis, are relevant for our understanding and elucidation of the initial steps underlying *SMAD4*-driven intestinal tumorigenesis.

## Background

Haploinsufficiency is defined as the condition where mutation or loss of a single allele is sufficient to alter the phenotype of a diploid cell [1]. Haploinsufficiency at a tumor suppressor locus may overcome the need for somatic loss or mutation of its wild-type allele, predicted as the rate-limiting event for tumor development by the Knudson's 'two-hit' model [2]. To date, experimental evidence for haploinsufficiency in cancer predispositions comes from the analysis of tumors obtained from mouse models or hereditary cancer patients carrying heterozygous *null *mutations at known tumor suppressor genes [3]. The absence of the second hit in a subset of these tumors has been attributed to many causes, including inactivation of the remaining allele by alternative mechanisms such as epigenetic silencing, mutations in non-coding sequences, or to limited sensitivity of the employed mutation detection protocol. However, *bona fide *haploinsufficiency has been demonstrated for a subset of tumor suppressor loci including *SMAD4 *[4-6], an intracellular mediator of the TGF-β and BMP signal transduction pathways [7,8]. Upon TGF-β or BMP signaling, SMAD4 binds to the receptor-activated SMADs and translocates to the nucleus where it modulates the transcription of a broad spectrum of target genes involved in cell growth inhibition, apoptosis, differentiation, and matrix production [7-9]. Somatic *SMAD4 *gene mutations are found in only a fraction of advanced sporadic colorectal cancers (CRCs) [10], whereas germline *SMAD4 *mutations are responsible for a subset of patients affected by juvenile polyposis syndrome (JPS; Online Mendelian Inheritance in Man 174900) [4], an autosomal dominant intestinal cancer syndrome. Although the original report showing that *SMAD4 *germline mutations are responsible for JPS also contained preliminary data indicating that loss of heterozygosity (LOH) of the wild-type allele occurred in a minority of the polyps examined [4], the most convincing evidence for haploinsufficiency at this locus came from the analysis of mouse models for juvenile polyposis. We and others showed that mice carrying targeted *Smad4 *mutations develop gastrointestinal (GI) polyps with initial retention of the wild-type *Smad4 *allele; complete functional loss only occurs at later stages of tumor progression within the epithelial compartment [5,6]. Notably, loss of a single *Smad4 *allele in the T-cell compartment and not in the intestinal epithelium resulted in mice characterized by hyperplasia and polyp formation in the GI tract, similar to the animals with constitutive *Smad4 *mutations [11]. These data indicate that Smad4 haploinsufficiency is likely to play a causative role in GI tumor formation by exerting a 'landscaping' effect from within the microenvironment as originally proposed by Kinzler and Vogelstein [12], and its complete loss of function in the epithelial cells at later tumor stages accompanies progression towards malignancy [5,6]. Whether *SMAD4 *causes polyp formation through haploinsufficiency has been challenged by two studies showing that the majority of tumors from JPS patients carrying germline mutations do show LOH at the wild-type locus [13,14]. These somatic events occurred both in the epithelial and stromal components of JPS polyps but not in the infiltrating lymphocytes [13].

Here, we have analyzed a cohort of juvenile polyps from patients with germline *SMAD4 *mutations for the retention of protein expression and confirmed that haploinsufficiency characterizes early stages of polyp formation in a substantial proportion of the cases. Moreover, we attempted the elucidation of the molecular basis of *Smad4 *haploinsufficiency by studying signal transduction and global gene expression in otherwise normal *Smad4*^+/- ^cells.

## Results

### SMAD4 haploinsufficiency underlies human juvenile polyposis

**Figure 1:**
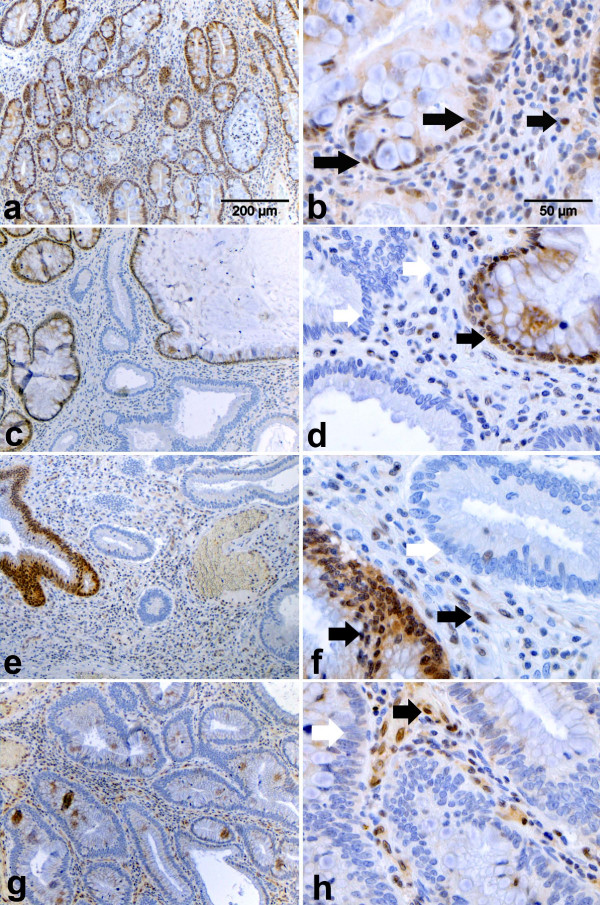
**Immunohistochemical analysis of SMAD4 protein expression in juvenile polyps from patients carrying *SMAD4 *germline mutations**. SMAD4 immunohistochemical analysis of hamartomatous polyps obtained from unrelated Juvenile Polyposis syndrome patients with established *SMAD4 *germline mutations (see Table 1). Images were taken at 10× (a, c, e, g,) and 40× (b, d, f, h). Filled (black) arrows indicate cells scored as positive, whereas white arrows point to negatives. **a-b**. Example of a polyp positive for SMAD4 nuclear staining in both the epithelial and stromal compartment (scored as double positive in Table 1). **c-d**. Polyp with heterogeneous SMAD4 expression pattern with patches of positively and negatively staining epithelial glands. Most of the stromal cells appear negative with few exceptions. This tumor was scored as +/- (epithelial) and – (stromal) in Table 1. **e-f**. Example of a juvenile polyp with heterogeneous SMAD4 expression in the epithelial compartment but with a more pronounced positive staining of the stromal tumor microenvironment. This tumor was scored as +/- (epithelial) and + (stromal) in Table 1. **g-h**. Example of a juvenile polyp characterized by negative SMAD4 staining throughout the epithelial cells. In the stromal compartment however, several positively staining cells are present. This tumor was scored as – (epithelial) and + (stromal) in Table 1.

Although haploinsufficiency has been thoroughly characterized in mouse models carrying targeted mutations in the *Smad4 *gene [5,6], two reports have shown that in the majority of tumors from JPS patients carrying *SMAD4 *germline mutations LOH could be detected at the wild-type allele [13,14]. To clarify this apparent discordance between mouse and man, and to establish whether *SMAD4 *behaves as a classical tumor suppressor gene or if, analogous to the *Smad4 *mouse models, haploinsufficiency underlies early stages of tumor formation in man, we performed SMAD4 immunohistochemistry (IHC) analysis of juvenile polyps from six unrelated JPS patients with known *SMAD4 *germline mutations (Table 1). Out of 13 polyps analyzed, three (23%) revealed a homogeneously negative staining of the epithelial tumor cells, thus indicating functional loss of the wild-type *SMAD4 *allele (Figure 1g and 1h). In the remaining cases, SMAD4 expression was either patchy with groups of negative glands scattered among positive ones (*n *= 6, 46%; Figure 1c to 1f), or showed clear retention of SMAD4 expression (*n *= 4, 31%; Figure 1a and 1b). As far as the tumor-associated stroma is concerned, different mesenchymal cell types, for example stromal fibroblasts and infiltrating lymphocytes, showed in almost all cases SMAD4 nuclear reactivity amidst a variable number of negative cells (Figure 1). However, no relationship could be established between the percentage of SMAD4-positive stromal cells and the loss/retention of expression in the adjacent epithelial glands. Also, although the total number of tumors analyzed is admittedly small, no correlation could be found between polyp size and loss/retention of SMAD4 expression.

**Table 1:** Results of the immunohistochemical analysis of intestinal polyps from Juvenile Polyposis syndrome patients carrying established *SMAD4 *germline mutations.

	***SMAD4 *Germline Mutation**		**Epithelial SMAD4 expression**	**Stromal SMAD4 expression**
	Nucleotide [ref.]	Amino Acid		
**JPS case #1**				
polyp A	nt 1042–43, 2 bp del, TTGTTA-TTTA [4]	FS 350X	**-**	**+**
polyp B	nt 1042–43, 2 bp del, TTGTTA-TTTA [4]	FS 350X	**-**	**-**
**JPS case #2**				
polyp A	nt 424+1, intron 2 TTGg-TTGa	splice defect	**+/-**	**+**
**JPS case #3**				
polyp A	nt 1058 TAC-TCC [40]	Tyr353Ser	**+/-**	**+**
polyp B	nt 1058 TAC-TCC [40]	Tyr353Ser	**+**	**+**
polyp C	nt 1058 TAC-TCC [40]	Tyr353Ser	**+**	**+**
**JPS case #4**				
polyp A	nt 533 TCA-TGA [40]	Ser178X	**+/-**	**+**
polyp B	nt 533 TCA-TGA [40]	Ser178X	**+**	**+**
**JPS case #5**				
polyp A	nt 687–692, 1 bp ins, TGGGGGGC- TGGGGGGGC [4]	FS 235X	**+**	**+**
polyp B	nt 687–692, 1 bp ins, TGGGGGGC- TGGGGGGGC [4]	FS 235X	**-**	**-**
polyp C	nt 687–692, 1 bp ins, TGGGGGGC- TGGGGGGGC [4]	FS 235X	**+/-**	**+/-**
**JPS case #6**				
polyp A	nt 1244–47, 4 bp del, AGACAGAG-AGAG [4]	FS 434X	**+/-**	**+**
polyp B	nt 1244–47, 4 bp del, AGACAGAG-AGAG [4]	FS 434X	**+/-**	**+**

In the last two columns, +, +/-, and - indicate homogeneously positive, patchy, and homogeneously negative nuclear SMAD4 staining, respectively (for examples of the various staining patterns, see Figure 1).

Overall, the IHC results confirm that in a substantial proportion of the polyps here analyzed from JPS patients with *SMAD4 *germline mutations, tumor onset does not follow Knudson's two-hit model as SMAD4 expression is retained in either all or in a considerable proportion of the epithelial tumor cells. In these cases, as previously shown in the *Smad4*^+/*E*6*sad *^mouse model [6], haploinsufficiency is likely to underlie juvenile polyp onset, whereas later stages of tumor progression are accompanied by loss of the wild-type allele.

### Smad4 haploinsufficiency results in partial inhibition of TGF-β and BMP signaling

In order to study the transcriptional and signal transduction defects arising from haploinsufficiency at the *Smad4 *tumor suppressor gene, we employed mouse embryonic stem (ES) cells where a *null *mutation, namely a single nucleotide deletion in the exon 6 splice acceptor site resulting in an unstable mRNA, is present in the endogenous locus [6,15]. The choice of ES cells as a cellular model to study *Smad4 *haploinsufficiency was made mainly based on observations that different components of the parenchymal and microenvironmental compartments are likely to contribute to polyp initiation and progression to malignancy [5,6,11], as described above. ES cells represent the inner cell mass of the pre-implantation blastocyst and thus precede the differentiation of the three main germ layers. Moreover, the employment of normal rather than neoplastic cells allows bypassing of confounders caused by the altered cellular physiology characteristic of tumor cells and focuses on the primary molecular and cellular consequences of *Smad4 *haploinsufficiency. The alternative use of mouse embryonic fibroblasts (MEFs) was made impossible by the early *in utero *lethality characteristic of the *Smad4*^*E*6*sad*/*E*6*sad *^embryos which precludes MEFs isolation with this genotype [15].

**Figure 2:**
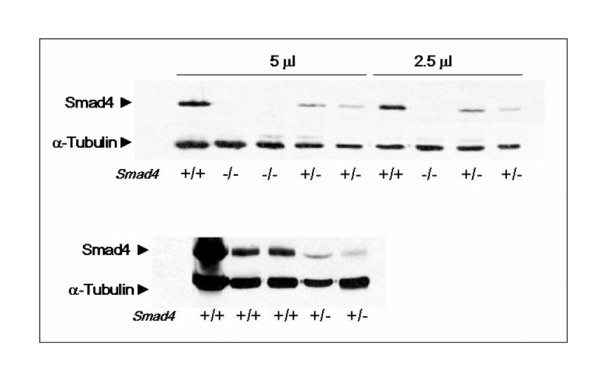
**Western analysis of *Smad4*-mutant embryonic stem cell lines**. SMAD4 western blot analysis demonstrates haploinsufficiency in embryonic stem (ES) and adult intestinal cells from *Smad4*^+/*E*6*sad *^mice. **Upper panel: **ES cell lysates loaded at two different protein amounts; **lower panel: **normal intestinal tissue lysates from *wild-type *and *Smad4*^+/*E*6*sad *^mice.

First, we evaluated Smad4 protein expression in wild-type (*Smad4*^+/+^), heterozygous (*Smad4*^+/*E*6*sad*^), and homozygous (*Smad4*^*E*6*sad*/*E*6*sad*^) ES cells obtained from pre-implantation blastocysts of interbred C57Bl6/J *Smad4*^+/*E*6*sad *^mice. To this aim, two independent hetero- and homozygous ES clones were analyzed by western blot. As shown in Figure 2 (upper panel), *Smad4*^*E*6*sad*/*E*6*sad *^cells did not reveal any protein expression thus confirming the *null *nature of this mutation, whereas heterozygous ES lines showed a consistent reduction in protein expression when compared with wild-type ES cells, indicative of their haploinsufficiency at the protein level. Moreover, western analysis of intestinal cells from *Smad4*^+/*E*6*sad *^mice validated the protein haploinsufficiency in adult tissues (Figure 2, lower panel).

**Figure 3:**
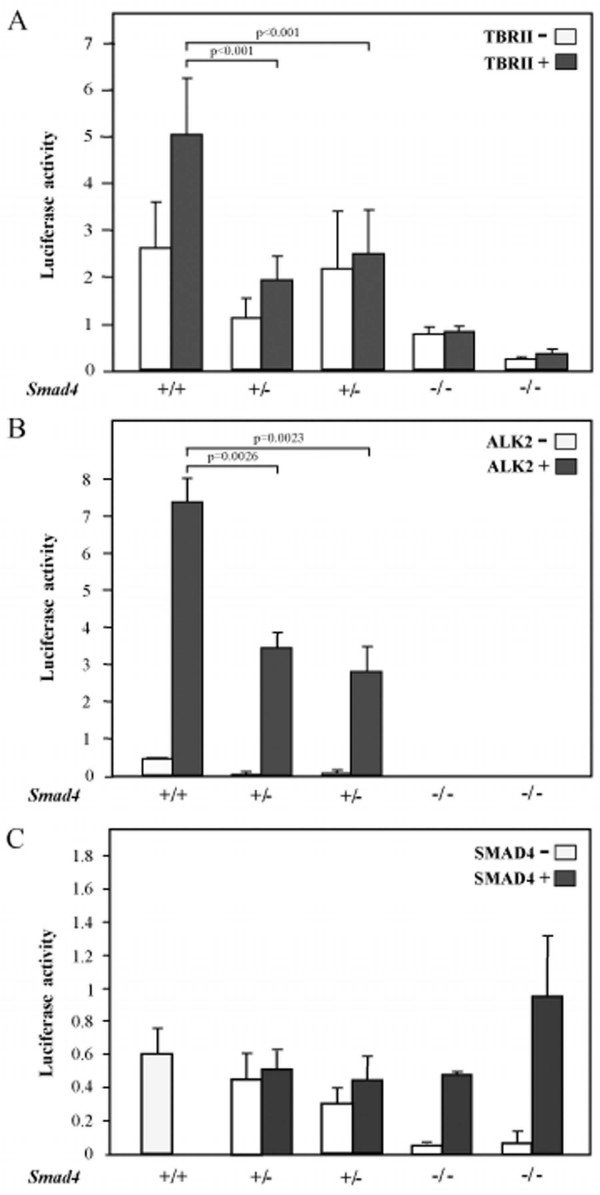
**TGF-β and BMP reporter assay analysis of *Smad4*-mutant embryonic stem cell lines**. TGF-β and BMP reporter assay analysis of *Smad4*-mutant embryonic stem (ES) cell lines. **A**. TGF-β (CAGA12-MLP-luciferase) and **B**. BMP (BRE-luciferase) reporter assays were carried out in *wild-type *(*+/+), Smad4*^+/*E*6*sad*^(+/-), and *Smad4*^*E*6*sad*/*E*6*sad *^(-/-) ES cell lines. Normalized CAGA-luc and BRE-luc levels are indicated for each cell line. For each genotype reporter activities are shown for cells transfected (TBRII+ and ALK2+) and non transfected (TBRII- and ALK2-) with the corresponding receptor-expressing vector. In **C**. the TGF-β reporter assays analysis was carried out after transfection with a *SMAD4*-expressing vector. Each bar represents the average of three independent experiments, and the error bars represent the standard deviation. For the CAGA12-MLP-luciferase assay, three independent ES wild-type clones have been employed. The WT bar represents the average of the luciferase activities measured in two independent experiments. p values were calculated by the statistical two samples *t*-test (two tails).

To determine whether the observed reduction of Smad4 expression in mouse *Smad4*^+/*E*6*sad *^ES and intestinal cells impairs TGF-β and BMP signaling, we measured the levels of the Smads transcriptional complex by a TGF-β reporter assay system [16]. Due to the insufficient expression levels of the type II TGF-β receptor (TBRII) in ES cells (data not shown), TGF-β reporter assay constructs were co-transfected with an expression vector (pCMV5-TBRII) encoding for the TBRII receptor [17]. A dose-response assay was subsequently performed which indicated that a TGFβ 1 concentration of 50 pmol provides the highest response in the TBRII-transfected cells (data not shown). As depicted in Figure 3A, *Smad4*^*E*6*sad*/*E*6*sad *^ES cells demonstrated a dramatic decrease in TGF-β signaling activity when compared with the wild-type controls. Notably, decreased TGF-β activity is already apparent in *Smad4*^+/*E*6*sad *^ES clones, characterized by an intermediate yet highly significant (*p *< 0.001) level of luciferase activity between wild-type and homozygous cells. The difference between *Smad4*^+/+ ^and *Smad4*^+/*E*6*sad *^ES cells was not significant when untransfected cells were employed.

Similar results were obtained by analyzing the *Smad4-*mutant ES cells for BMP signaling levels by the BRE-luc reporter assay transfected together with a constitutively active ActR-I receptor (Alk-2) [18]: while *Smad4*^*E*6*sad*/*E*6*sad *^ES cells showed a marked decrease in BMP signaling, haploinsufficient cells revealed an intermediate though significant (p = 0.0024) level between wild-type and Smad4-deficient ES cells (Figure 3B). Also, rescue of homozygous *Smad4*^*E*6*sad*/*E*6*sad *^ES cells by transfection with a human *SMAD4 *expression vector [9] fully restored TGF-β signaling (Figure 3C).

### Smad4 affects genome-wide gene expression in a dosage-dependent fashion

In order to further characterize the effects of Smad4 haploinsufficiency on gene transcription, expression profiling analysis of total RNA samples from wild-type, *Smad4*^+/*E*6*sad*^, and *Smad4*^*E*6*sad*/*E*6*sad *^ES cells was performed using the Affymetrix MOE430 2.0 array. Two independent clones for each genotype were employed for the analysis. Two individual lists of differentially expressed genes were generated by applying a p-value threshold of *p *< 0.01 and *p *< 0.05 for the homo- and heterozygous cells, respectively. Comparison of these two data sets led to the identification of a signature of 79 differentially expressed genes (31 up- and 48 down-regulated respectively) common to *Smad4 *hetero- and homozygous cells, 64 of which (24 up- and 40 down) represent functionally annotated genes (Additional file 1).

**Figure 4:**
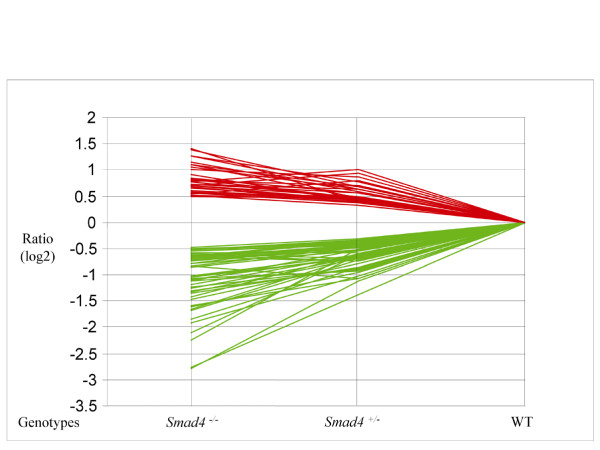
**Trend analysis of *Smad4 *dosage-dependent transcriptional targets**. Trend analysis of the 79 genes found to be differentially expressed in both *Smad4*^+/*E*6*sad *^and *Smad4*^*E*6*sad*/*E*6*sad *^embryonic stem cells. The fold changes are represented as (log2) ratio values and plotted as red and green lines for up- and down-regulated genes, respectively.

Among the differentially expressed entries, a broad spectrum of functional categories is represented: members of known signal transduction pathways (for example, *Mdm2*, *Axin2*, *Smad7*, *Zak*), growth factors (*Fgf5*, *Fgf8*, *Igfbp3*, *Lefty2*), immunity related genes (*Irgm*, *Il23a*, *Cxcl14*), and transcription factors (*Nrip1*, *Eomes*, *Stat3*, *Cnot6*, *T *brachyury homolog) (Additional file 1). This is also confirmed by Ingenuity Pathway analysis performed on the 64 gene signature (Additional file 2). The presence of *Smad4 *itself within the list of differentially expressed genes serves as an internal confirmation of the validity of our approach. Notably, when the fold changes levels relative to these 79 genes are plotted in a trend analysis according to the *Smad4 *genotypes, the Smad4 dosage-dependent gradient of transcriptional response becomes apparent (Figure 4).

**Figure 5:**
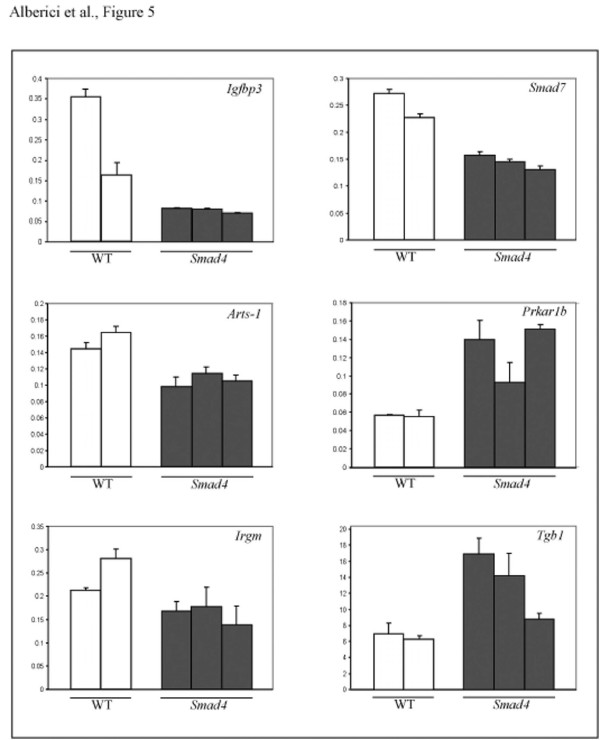
**qPCR validation of the expression profiling results**. Quantitative PCR analysis of a selection of six genes differentially regulated in *Smad4*^+/*E*6*sad *^cells. Gene expression was quantified in normal intestinal tissues from two *wild-type (WT) *and three *Smad4*^+/*E*6*sad *^(*Smad4*) animals and is plotted as ratio over the reference gene (see Materials and Methods). Each *bar *represents the average of three independent experiments.

In order to validate the observed Smad4 dosage-dependent effect on the transcriptional regulation of a subset of target genes, we performed quantitative real-time RT-PCR on laser-capture microdissected (LCM) normal intestinal cells obtained from two wild-type and three *Smad4*^+/*E*6*sad *^animals. From the list of 64 genes differentially expressed upon Smad4 haploinsufficiency, we selected six known to be expressed in the GI tract and characterized by at least a two-fold change in the ES cell expression profiling data (log2>1). As an internal reference standard, we employed the *Cryzl *(crystallin zeta quinone reductase-like 1) gene, which retains constant expression levels between all ES and adult intestinal cells (data not shown). Similar to the ES cells expression profiling results, both up- (*Prkar1b *and *Tgb1*) and down-regulated (*Smad7, Irgm, Arts-1 *and *Igfbp1*) genes showed consistent changes in gene expression levels in *Smad4*^+/*E*6*sad *^normal intestinal cells when compared with intestinal epithelia from wild-type (*Smad4*^+/+^) animals (Figure 5), thus validating the microarray expression profiling results.

## Discussion

Over the last few years it has become clear that in a subset of tumor suppressor genes the somatic inactivation or mutation of the wild-type allele (the second hit) does not invariably represent the rate-limiting tumor formation step [1,3,19]. It is generally thought that haploinsufficiency may affect normal cell function and homeostasis, possibly in a synergistic manner with other genetic or epigenetic somatic hits at unrelated cancer genes. Both JPS patients and mouse models carrying loss of function mutations at the *SMAD4 *tumor suppressor gene represent illustrative examples of haploinsufficiency in GI tract tumorigenesis. In partial disagreement with Knudson's two-hit model, somatic loss of the wild-type allele is not a rate-limiting event in intestinal polyp formation in *Smad4*-mutant mouse models [5,6]. Accordingly, as shown in our study, the majority of polyps from JPS patients carrying *SMAD4 *germline mutations retain SMAD4 expression both in epithelial tumor cells and in their stromal microenvironment, thus indicating haploinsufficiency. Previously, Howe et al showed that juvenile polyps from *SMAD4 *mutation carriers reveal loss of the wild-type allele in only 9% (1/11) of their cases [4]. However, the analysis was done by an exon-specific PCR assay unable to detect more subtle somatic hits such as point mutations and epigenetic silencing. Notably, two subsequent and more thorough reports have shown by LOH, fluorescence in situ hybridization, and IHC analysis that loss of the wild-type allele could be detected in the majority of tumors from JPS patients carrying *SMAD4 *germline mutations [13,14]. Moreover, SMAD4 loss was observed in both epithelial and some of the stromal cells, which was interpreted by the authors as an indication of the clonal origin of these lesions, and of the fact that *SMAD4 *represents a classical 'gatekeeper' tumor suppressor rather than a 'landscaper' as originally proposed [12,13]. How can the apparent discordance between the present study and the reports by Woodford-Richens and colleagues be solved? From the IHC analysis depicted in Figure 1 it should be clear that a high degree of heterogeneity in SMAD4 expression characterizes juvenile polyps both in the epithelial and mesenchymal compartments. This heterogeneity, when reduced to more quantitative values as in the case of PCR-based LOH analysis of whole tumor specimens comprehensive of both parenchymal and microenvironmental cells, may result in loss of accuracy. Also, differences in interpretation of IHC images may partly underlie this discrepancy. In their IHC analysis of juvenile polyps from *SMAD4*-mutant JPS patients [14], Woodford-Richens et al present an example where, similar to observations in our study, a heterogeneous staining pattern is observed with positive epithelial glands amidst negative ones. Last but not least, both the two-hit and haploinsufficiency models appear to hold true for *SMAD4*-driven tumorigenesis, and this may depend on the molecular nature and pathogenicity of the first hit, namely the germline mutation. As predicted by the 'just right' model for the *APC *tumor suppressor gene [20,21], the molecular nature of the first hit at a tumor suppressor locus affects the type of second-hit mutation at the wild-type allele. It is plausible to think that while some *SMAD4 *mutations require functional inactivation of the wild-type allele to trigger tumor formation, others can result in juvenile polyp onset without this otherwise rate-limiting somatic step.

A second important aspect is relative to the role of the *SMAD4 *in tumor formation either as an epithelial 'gatekeeper' or as a 'landscaper', that is, acting from within the microenvironment to affect epithelial homeostasis [12]. Recently, it was shown that selective loss of *Smad4 *in the mouse T-cell compartment results in intestinal adenomas reminiscent of JPS polyps [11]. Notably, loss of a single *Smad4 *allele in T-cells also resulted in hyperplasia and polyp formation in the intestinal epithelial layer, thus indicating that Smad4 haploinsufficiency plays a causative role in GI tumor formation by exerting a 'landscaping' effect from within the stromal compartment [11]. Conversely, our own observation, according to which more advanced lesions of the *Smad4*^+/*E*6*sad *^mouse model show complete loss of Smad4 expression [6], is indicative of an additional role for the complete loss of Smad4 function in the epithelial compartment at later tumor progression stages. Whether the same holds true for *SMAD4*-driven juvenile polyp formation in man is still debatable. From our own IHC analysis (Figure 1), it should be evident that the SMAD4 expression pattern in the tumor microenvironment appears rather heterogeneous, with a mixture of positive and negative stromal fibroblasts and infiltrating lymphocytes. However, these results are inconclusive in discriminating between the 'gatekeeper' versus 'landscaper' scenarios. In fact, there is little doubt about the active role played by the tumor microenvironment, especially in the presence of a TGF-β signaling defect. Previous reports have shown that loss of function mutations at the *TBRII *gene can both trigger epithelial tumorigenesis from the stromal layer [22] and underlie malignant transformation when induced in the parenchymal cells of intestinal adenomas initiated by *Apc *mutations [23]. Similar observations were made for the *LKB1 *gene (also known as *STK11*), responsible for Peutz-Jeghers syndrome (PJS; OMIM 175200), an autosomal dominant predisposition to hamartomas (polyps of the GI tract with a pronounced mesenchymal component, very similar to those characteristic of JPS). *Lkb1*^+/- ^mice develop intestinal polyps which often retain the wild type allele [24]. Accordingly, LOH is not an obligate step in polyps from PJS patients with germline *LKB1 *mutations [25]. Notably, monoallelic loss of murine *Lkb1 *in the smooth muscle compartment results in GI polyps indistinguishable from those observed in mice and in PJS patients with a constitutive mutation, thus confirming the 'landscaping' role of *Lkb1 *haploinsufficiency [26]. Further molecular analysis of these mice revealed that the partial loss of Lkb1 function results in a TGF-β signaling defect within the stromal compartment likely to contribute to polyp formation by generating a permissive microenvironment for the malignant transformation of the epithelium [26]. Given the heterogeneous composition of the tumor microenvironment comprising not only stromal fibroblasts but also smooth muscle cells and various cellular types of immune origin, it is plausible to think that haploinsufficiency at members of the TGF-β signaling pathways such as *SMAD4 *and *LKB1 *affects cell to cell communication in different tissues, thus leading to loss of tissue architecture.

In this study, we have shown that *Smad4 *haploinsufficiency results in dosage-dependent inhibition of TGF-β and BMP signaling, thus affecting epithelial cell proliferation and differentiation both in the stromal and epithelial compartments and likely to underlie juvenile polyp formation in the intestinal tract. Also, expression profiling of *Smad4 *haploinsufficient cells revealed the existence of a subset of target genes whose expression is specifically regulated by decreased dosages of this tumor suppressor, presumably through TGF-β and BMP signaling, as also shown by the differential expression of two known downstream targets of the TGF-β pathway, namely *Smad7 *and *Tgfb1*. *Smad7 *is both an inhibitor of TGF-β signaling and itself a TGF-β downstream target [27]. Hence, *Smad7 *down-regulation, as observed in the *Smad4*^+/*E*6*sad *^ES cells, reflects the observed TGF-β signaling inhibition. Tgfb1 is an extracellular protein with only poorly characterized functions, though its significant over-expression, as observed in *Smad4*-mutant ES cell lines, has also been reported in sporadic CRC [28].

Among the genes shown to be differentially up- or down-regulated in a Smad4 dosage-dependent fashion, members of other signal transduction pathways were also included (Additional files 1 and 2). *Mdm2 *up-regulation, as observed in *Smad4*^+/*E*6*sad *^cells, may favor tumor transformation by inhibiting p53-mediated transactivation [29] and by destabilizing retinoblastoma (RB). The gene encoding for the scaffold protein Axin2 (conductin), down-regulated in *Smad4*^+/*E*6*sad *^cells, has been previously implicated in canonical Wnt signaling and colorectal pathogenesis [30]. The latter is indicative of cross-talking between TGF-β and Wnt signal transduction already in haploinsufficiency. Down-regulation of the *Igfbp3 *gene, encoding for the insulin growth factor binding protein, in *Smad4*^+/*E*6*sad *^ES and intestinal cells may also represent a relevant early step in tumor formation. *Igfbp3 *has been described as tumor suppressor gene [31], due to its role in the regulation of cell proliferation and apoptosis, and its differential methylation in a substantial fraction of CRC cases [32].

Overall, Smad4 haploinsufficiency affects both TGF-β and BMP signaling together with a broad spectrum of transcriptional targets and cellular functions. Future studies will reveal which of these target genes are preferentially affected in a specific cellular compartment, and the paracrine or cell autonomous effect that they exert on epithelial tumorigenesis.

## Conclusion

In this study we have shown that haploinsufficiency at the *SMAD4 *tumor suppressor locus underlies polyp formation in a proportion of GI tumors from JPS patients, as previously shown in *Smad4*-mutant mouse models. Moreover, *SMAD4 *haploinsufficiency affects both the TGF-β and BMP signal transduction pathways together with a broad spectrum of transcriptional targets and cellular functions. These results contribute to our understanding of the cellular and molecular mechanisms underlying intestinal tumorigenesis due to TGF-β (and BMP) signaling defects not only in the parenchymal cells but also from within the stromal microenvironment. This is of fundamental but also of clinical relevance as these dosage-specific transcriptional targets may offer novel opportunities in the development of tailor-made therapeutic strategies.

## Methods

### Generation of Smad4-mutant ES cell lines

*Smad4*^+/*E*6*sad *^mice, available on the inbred C57Bl/6J background, were inter-bred and the resulting blastocysts harvested at 3.5 dpc. Flushed pre-implantation blastocysts were then individually cultured on 96-well dishes coated with MEFs as previously described [33].

All mouse experiments were performed upon approval of the local animal experiment committee (DEC permissions nr. EUR 596, 600, 623 and 730) and according to internationally recognized guidelines (as described by the *Code of Practice Dierproeven in het Kankeronderzoek*).

### Smad4 western analysis

Equal amounts (40 μg) of protein lysates were separated on 12% SDS polyacrylamide gels, and further subjected to immunoblotting according to standard procedures. Several studies have validated the specificity and sensitivity of the B-8; sc-7966 monoclonal antibody against SMAD4 (Santa Cruz Biotechnology) to detect alterations of protein expression in both mouse and human specimens. The B-8; sc-7966 primary antibody was employed for western analysis at a 1:100 dilution. Peroxidase-conjugated secondary antibodies (Jackson Immunoresearch) were visualized with an enhanced chemiluminescence system.

### Reporter assay analysis

ES cells grown on tissue culture dishes coated by mitotically inactivated primary MEFs were transfected by Lipofectamine 2000 (Life Technologies) with 250 ng of the reporter plasmid ((CAGA)12-MLP-luciferase for the TGF-β signaling or BRE-luciferase for the BMP signaling), 100 ng of receptor-expressing vector (TGFBRII or, for BMP signaling, a constitutively active form of ALK2) [17,18] (all kindly provided by Professor P ten Dijke), and 5 ng of a *Renilla reniformis *luciferase-expressing vector. For the rescue experiments, 100 ng of the *Smad4*-pCMV5 expression vector [9] were transfected together with (CAGA) 12-MLP-luciferase and TGFBRII. After 24 hours, ES cells transfected with the (CAGA)12-MLP-luciferase were stimulated with recombinant human TGF-β (50 pmol) for 1 hour before measuring luciferase activity with the luminometer Fluoroskan Ascent CF (Labsystems) using the Dual Luciferase Reporter Assay system (Promega). Luciferase activities were calculated as a ratio between the specific luciferase-reporter construct and the Renilla luciferase levels, for a total of three different experiments, each carried out in triplicate. For the CAGA12-MLP-luciferase assay, three independent ES wild-type clones were used and the average of their luciferase activities measured in two independent experiments.

### Immunohistochemical analysis

Formalin-fixed, paraffin-embedded intestinal polyps were prepared as 4 μm sections and immunostained with the mouse Smad4 B-8; sc-7966 monoclonal antibody directed against Smad4 (Santa Cruz Biotechnology Inc, dilution 1:100). After antigen retrieval treatment (10 min boiling in Tris-EDTA pH 8.0), endogenous peroxidase was inactivated with 1% H_2_O_2_/PBS. A 30 min pre-incubation step in 5% non-fat dry milk in PBS was followed by incubation with the Smad4 antibody overnight at 4°C in pre-incubation buffer. Sections were then stained with the Envision HRP-ChemMate kit (DAKO). Smad4 IHC staining was evaluated after brief hematoxylin counterstaining of the slides.

### Expression profiling analysis

Total RNA was labeled using the GeneChip One-Cycle Target Labeling and Control Reagents kit, and hybridized to MOE430 2.0 Affymetrix oligonucleotide arrays, according to the manufacturers' instructions. Raw signal intensities were extracted and summarized from cel-files, followed by normalization using the robust multi-array average expression measure implemented in the Bioconductor package affylmGUI [34]. No filtering was applied to the data. A Bayesian linear regression model was used to detect differentially expressed genes implemented in the Bioconductor package limma [35,36]. All Bioconductor packages were used with R statistical Computing Software v2.2.1 [37].

### Quantitative real-time RT-PCR analysis

LCM of intestinal tissues (approx. 6000 cells) was performed as previously described [38]. PCR analysis was carried out in triplicate in 25 μl volumes using 1 μl of cDNA and SYBR^® ^Green Dye (Applied Biosystems) on the MyiQ Single-Color Real-Time PCR Detection System (Bio-Rad). Primers sequences are listed below. Standard curves for the target genes and the reference *Cryzl1 *gene were generated and the normalization and ratio were calculated as described [39], (see Table 2).

**Table 2:** Primers used in real-time RT-PCR analysis

**Gene**	**Forward Primer**	**Reverse Primer**
*Cryzl1*	5'-AGCTGCTGGCGTCATCCG-3'	5'-CTGTGGTGGGCTAACTGAATGG-3'
*Smad4*	5'-GTGACTGTGGATGGCTATGTGG-3'	5'-GCAACCTCGCTCTCTCAATCG-3'
*Arts-1*	5'-GCAGACTTGGACAGATGAAGG-3'	5'-TGACTTCCACTCTCTGAAATAGC-3'
*Smad7*	5'-TGCCTCGGACAGCTCAATTCG-3'	5'-CCCACACGCCATCCACTTCC-3'
*Prkar1b*	5'-GCCCGAATCCCTGTCCCTTG-3'	5'-TGGCTGGCTCATATCACACTCC-3'
*Irgm*	5'-ACAGGCTCCAGCAGGTTACC-3'	5'-TTGCCACAGTCTCCTTGATTCC-3'
*Tgfb1*	5'-CAAACAGGCGTCAGCGTATTCC-3'	5'-GGCTCTCCTCCTCGGTCTTCC-3'
*Igfbp1*	5'-CCCAGAGGCGTCCACATCC-3'	5'-GTCCACACACCAGCAGAAGC-3'

## Competing interests

The authors declare that they have no competing interests.

## Authors' contributions

PA was responsible for the immunohistochemistry, reporter assay, western and Q-PCR analyses, and has been involved in drafting the manuscript. CG performed the microarray analysis and the bioinformatic analysis of the resulting data. PF was mainly responsible for the histological processing of the tumor tissues and the SMAD4 immunohistochemistry analysis. MMG contributed to the target validation by quantitative PCR. IdV carried out the TGF-β reporter assays. RJS, AR, and LAA contributed the intestinal polyps from JPS patients with SMAD4 germline mutations. RF designed and supervised the study and wrote the final manuscript. All authors have read and approved the final manuscript.

## Supplementary Material

Additional file 1 Supplementary Table 1. List of 64 functionally annotated genes differentially express in *Smad4*^+/*E*6*sad *^and *Smad4*^*E*6*sad*/*E*6*sad *^ES cell lines. Expression profiling values are expressed as absolute fold change values when compared to *Smad4*^+/+ ^ES cells. Click here for file

Additional file 2 Supplementary Table 2. Ingenuity Pathway Analysis of the 64 functionally annotated genes differentially expressed (denoted as "focus molecules" in bold) in *Smad4*^+/*E*6*sad *^and *Smad4*^*E*6*sad*/*E*6*sad *^ES cell lines. The column denoted as "Top Functions" describe the gene ontology groups to which the genes encompassed in a given Ingenuity Network belong. Only the top 4 networks with the most significant scores are included. Click here for file
