# Supplementary material for: Smad4 haploinsufficiency: a matter of dosage
Source: Pathogenetics. 2008 Nov 3;1:2. doi: 10.1186/1755-8417-1-2 (PMC2580039; doi:10.1186/1755-8417-1-2)
Supplement: Additional file 1 — Supplementary Table 1. List of 64 functionally annotated genes differentially express in Smad4+/E6sad and Smad4E6sad/E6sad ES cell lines. Expression profiling values are expressed as absolute fold change values when compared to Smad4+/+ ES cells. [file 1755-8417-1-2-S1.doc]

## Supplementary Table 1. List of 64 functionally annotated genes differentially express in *Smad4*^+/E6sad^ and *Smad4*^E6sad/E6sad^ ES cell lines. Expression profiling values are expressed as absolute fold change values when compared to *Smad4*^+/+^ ES cells.

| Gene Symbol and Name | **Unigene** | **Fold Change**  ***Smad4*^E6sad/E6sad^*** | **Fold Change**  ***Smad4* ^+/E6sad^*** |
| --- | --- | --- | --- |
| ***Immunity Response*** |  |  |  |
| ARTS-1 (type 1 tumor necrosis factor receptor shedding aminopeptidase regulator) | Mm.83526 | -2.8 | -1.7 |
| CXCL14 (chemokine (C-X-C motif) ligand 14) | Mm.30211 | 1.7 | -1.9 |
| GBP6 (guanylate binding protein family, member 6) | Mm.45740 | -2.3 | -1.8 |
| IL23A (interleukin 23, alpha subunit p19) | Mm.125482 | 1.5 | 1.3 |
| IRGM (immunity-related GTPase family, M) | Mm.29938 | -3.8 | -2 |
| TAPBP (TAP binding protein ,tapasin) | Mm.392082 | -1.7 | -1.4 |
| ***Molecular Transport*** |  |  |  |
| ATP11A (ATPase, Class VI, type 11A) | Mm. 257837* | 1.8 | 1.5 |
| LMAN1 (lectin, mannose-binding, 1) | Mm.290857 | -2.5 | -1.9 |
| SERPINH1 (serpin peptidase inhibitor, clade H (heat shock protein 47), member 1) | Mm.22708 | 1.8 | 1.4 |
| STEAP1(six transmembrane epithelial antigen of the prostate 1) | Mm.85429 | 1.7 | 1.6 |
| CYP26A1 (cytochrome P450, family 26, subfamily A, polypeptide 1) | Mm.42230 | -1.5 | -2 |
| ***Signal Transduction*** |  |  |  |
| AXIN2 (axin 2, conductin, axil) | Mm.71710 | -1.5 | -1.7 |
| COMMD3 (COMM domain containing 3) | Mm.249586 | 2.2 | 1.6 |
| FGF5 (fibroblast growth factor 5) | Mm.5055 | -6.8 | -2.6 |
| FGF8 (fibroblast growth factor 8,androgen-induced) | Mm.4012 | -2.5 | -1.6 |
| GPR124 (G protein-coupled receptor 124) | Mm.87046 | 2 | 1.8 |
| IGFBP3 (insulin-like growth factor binding protein 3) | Mm.29254 | -3.1 | -1.9 |
| MDM2 (Mdm2, transformed 3T3 cell double minute 2, p53 binding protein) | Mm.22670 | 1.5 | 1.3 |
| PARD6G (par-6 partitioning defective 6 homolog gamma) | Mm.24678 | 1.7 | 1.4 |
| PRKAR1B (protein kinase, cAMP-dependent, regulatory, type I, beta) | Mm.306163 | 2.6 | 1.6 |
| PTDSR (phosphatidylserine receptor) | Mm.383423 | -1.6 | -1.3 |
| PTPRN2 (protein tyrosine phosphatase, receptor type, N polypeptide 2) | Mm.206054 | -1.6 | -1.3 |
| RHOF (ras homolog gene family, member F (in filopodia) | Mm.253876 | -1.6 | -1.3 |
| SH3BP5 (SH3-domain binding protein 5 (BTK-associated) | Mm.383198 | 1.7 | 1.5 |
| SMAD4 (SMAD, mothers against DPP homolog 4 (Drosophila) | Mm.100399 | -2.7 | -1.5 |
| SMAD7 (SMAD, mothers against DPP homolog 7 (Drosophila) | Mm.34407 | -3.6 | -1.8 |
| STAT3 (signal transducer and activator of transcription 3) | Mm.249934 | -1.7 | -1.4 |
| TULP4 (tubby like protein 4) | Mm.28251 | 1.7 | 1.5 |
| ***Development*** |  |  |  |
| T (T, brachyury homolog, in mouse) | Mm.913 | -7 | -2.2 |
| ODZ4 (odz, odd Oz/ten-m homolog 4 in Drosophila) | Mm.254610 | 1.4 | 1.4 |
| ZIC5 (Zic family member 5 (odd-paired homolog, Drosophila) | Mm.390761 | -2 | -1.6 |
| ***Transcription and Translation Regulation*** |  |  |  |
| CNOT6 (CCR4-NOT transcription complex, subunit 6) | Mm.247113 | -1.6 | -1.4 |
| EIF4E2 (eukaryotic translation initiation factor 4E member 2) | Mm.227183 | -1.4 | -1.3 |
| EOMES (eomesodermin homolog, in Xenopus laevis) | Mm.200692 | -2.1 | -1.9 |
| NRIP1 (nuclear receptor interacting protein 1) | Mm.390915 | 1.5 | 1.4 |
| RARA (retinoic acid receptor, alpha) | Mm.103336 | -1.5 | -1.3 |
| ZFP28 (zinc finger protein 28 homolog, in mouse) | Mm.127014 | 1.5 | -1.3 |
| ZNF524 (zinc finger protein 524) | Mm.19974 | -1.4 | -1.3 |
| ZNRF3 (zinc and ring finger 3) | Mm.216313 | -1.6 | -1.7 |
| ***Metabolism*** |  |  |  |
| PSMB8 (proteasome (prosome, macropain) subunit, beta type, 8) | Mm.180191 | -3.1 | -1.6 |
| RPL17 (ribosomal protein L17) | Mm.276337 | -4.7 | -1.4 |
| TRIM12 (tripartite motif protein 12) | Mm.327033 | -2.1 | -1.6 |
| CYP26A1 (cytochrome P450, family 26, subfamily A, polypeptide 1) | Mm.42230 | -2 | -1.5 |
| GALNT10 (UDP-N-acetyl-alpha-D-galactosamine:polypeptide N-acetylgalactosaminyltransferase 10) | Mm.271670 | -1.7 | -1.5 |
| SEPP1 (selenoprotein P, plasma, 1) | Mm.392203 | -1.5 | 1.3 |
| ENPP3 (ectonucleotide pyrophosphatase/phosphodiesterase 3) | Mm.338425 | -1.5 | 1.3 |
| HERC5 (hect domain and RLD) | Mm. 297393 | -3 | -2.1 |
| ***Blood coagulation*** |  |  |  |
| ANXA8 (annexin A8) | Mm.3267 | 1.4 | 1.7 |
| ***Cell Proliferation*** |  |  |  |
| DERL2 (Der1-like domain family, member 2) | Mm.28131 | -1.4 | -1.2 |
| LEFTY2 (left-right determination factor 2) | Mm.87078 | -4.3 | -1.5 |
| PEG10 (paternally expressed 10) | Mm.320575 | 1.4 | 1.3 |
| ***Cell Adhesion*** |  |  |  |
| CYR61 (cysteine-rich, angiogenic inducer, 61) | Mm.1231 | -1.8 | -2.1 |
| TGFBI (transforming growth factor, beta-induced, 68kDa) | Mm.14455 | 2.2 | 1.4 |
| ***Cell Cycle*** |  |  |  |
| PMP22 (peripheral myelin protein 22) | Mm.1237 | 2.6 | 1.3 |
| ZAK (sterile alpha motif and leucine zipper containing kinase AZK) |  | 1.6 | 1.4 |
| ***Cytoskeleton*** |  |  |  |
| ENC1 (ectodermal-neural cortex (with BTB-like domain) | Mm.241073 | -1.4 | -1.3 |
| ***Unknown*** |  |  |  |
| DDIT4L (DNA-damage-inducible transcript 4-like) | Mm.250841 | 2.4 | 1.6 |
| DENND1C (DENN/MADD domain containing 1C) | Mm.284447 | -1.5 | -1.6 |
| GBP4 (guanylate binding protein 4) | Mm. 45740 | -2.2 | -1.4 |
| KIAA0738 (KIAA0738 gene product) | Mm.24652 | 1.6 | 1.3 |
| KLHL26 (kelch-like 26 , in Drosophila) | Mm.187090 | 1.8 | 1.3 |
| NOPE (likely ortholog of mouse neighbor of Punc E11) | Mm.209041 | 1.7 | 2 |
| PHF19 (PHD finger protein 19) | Mm.65691 | -2.1 | -1.6 |
| PLEKHG2 (pleckstrin homology domain containing, family G member 2) | Mm.235700 | -1.6 | -1.4 |
| PSORS1C2 (psoriasis susceptibility 1 candidate 2) | Mm.34201 | -2.3 | -1.5 |
