# Supplementary material for: Smad4 haploinsufficiency: a matter of dosage
Source: Pathogenetics. 2008 Nov 3;1:2. doi: 10.1186/1755-8417-1-2 (PMC2580039; doi:10.1186/1755-8417-1-2)
Supplement: Additional file 2 — Supplementary Table 2. Ingenuity Pathway Analysis of the 64 functionally annotated genes differentially expressed (denoted as "focus molecules" in bold) in Smad4+/E6sad and Smad4E6sad/E6sad ES cell lines. The column denoted as "Top Functions" describe the gene ontology groups to which the genes encompassed in a given Ingenuity Network belong. Only the top 4 networks with the most significant scores are included. [file 1755-8417-1-2-S2.doc]

Supplementary Table 2. Ingenuity Pathway Analysis of the 64 functionally annotated genes differentially expressed (denoted as “focus molecules” in bold) in *Smad4*^+/E6sad^ and *Smad4*^E6sad/E6sad^ ES cell lines. The column denoted as “Top Functions” describe the gene ontology groups to which the genes encompassed in a given Ingenuity Network belong. Only the top 4 networks with the most significant scores are included.

| ID | Molecules in Network | **Score** | **Focus Molecules** | **Top Functions** |
| --- | --- | --- | --- | --- |
| **1** | BCL2L1, **C14ORF46**, **DDIT4L**, ELA2, **EOMES**, EPAS1, ERBB2, EZH2, FOXA2, FRAP1, **GALNT10**, **GBP6**, IGFBP1, **IGFBP3**, IGFBP4, IGFBP5, IL4, IL6, **IL23A**, IL6R, INHBA, **LEFTY2**, MMP7, **NRIP1**, **PHF19**, **PSMB8**, PSME1, PSME2, **PTDSR**, **RARA**, **RHOF**, **STAT3**, TF, TNFRSF1A, VHL | 26 | 15 | Cancer, Cellular Growth and Proliferation, Cell Death |
| **2** | **ANXA8**, BAX, BGLAP, COL2A1, **CYR61**, ERBB2, **GBP4**, IFNG, **IGFBP3**, IGFBP4, INHBA, **IRGM**, KLF3, KLF4, KLF6, MMP2, MMP3, MYCN, NF2, NFATC2, **PARD6G**, **PLEKHG2**, **PRKAR1B**, RAC1, **SEPP1**, **SERPINH1**, SFTPC, SMARCA4, SPP1, **TAPBP**, TGFB1, **TGFBI**, THBS1, TNFRSF1A, TP53 | 19 | 12 | Cancer, Tumor Morphology, Cellular Movement |
| **3** | ACTA2, ANXA2, **ARTS-1**, **AXIN2**, CASP8, CD44, CDH3, CTNNB1, **ENC1**, ERBB2, FGF3, **FGF8**, FZD8, **HERC5**, IL6R, INHBA, LDB1, MMP7, MSX1, MYC, MYCN, NFYB, **NOPE**, PAX5, **PEG10**, PLAT, RB1, **RPL17**, T, TGFBR1, TGFBR2, THBS1, VPS39, **ZAK**, **ZNF524** | 17 | 11 | Developmental Disorder, Cancer, Tumor Morphology |
| **4** | BGLAP, CLEC11A, COL2A1, COL7A1, **COMMD3**, **CXCL14**, **CYP26A1**, EGR2, ERBB2, FGF2, **FGF5**, FSHR, HOXC8, IGFBP1, **IGFBP3**, IGFBP4, IGFBP5, KLKB1, **LMAN1**, **MDM2**, MPZ, MYCN, NFKB1, PLAT, **PMP22**, PRKACA, **PTPRN2**, RUNX1, SAT, **SMAD4**, **SMAD7**, SP1, STAMBP, TF, WT1 | 17 | 11 | Cancer, Cellular Growth and Proliferation, Cell Death |
